# Supplementary material for: The Anti-Inflammatory Effect of Carrageenan/Echinochrom Complex at Experimental Endotoxemia
Source: Int J Mol Sci. 2022 Oct 3;23(19):11702. doi: 10.3390/ijms231911702 (PMC9570226; doi:10.3390/ijms231911702)

Supplementary materials

Figure S1. Chemical structures of the repeating units of κ-CRG

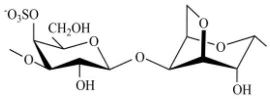

Figure S2. Structure of Ech A (Ech A, 6-ethyl-2,3,5,7,8-pentahydroxy-1,4-naphthoquinone)

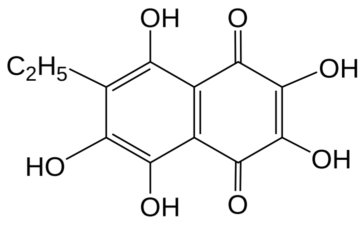

Figure S3. Cytotoxic activity of CRG+Ech (A) and Ech (B) against RAW 264.7 cells (2 × 104 cells/well).

Incubation time 24h.

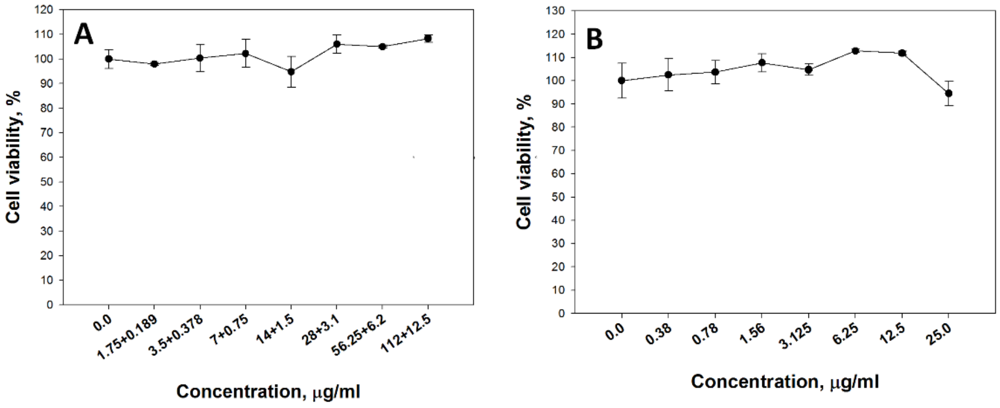

Supplement: Supplementary file 1 [file ijms-23-11702-s001.zip › ijms-1904173-supplementary.pdf]
